# Parakeet: a digital twin software pipeline to assess the impact of experimental parameters on tomographic reconstructions for cryo-electron tomography

**DOI:** 10.1098/rsob.210160

**Published:** 2021-10-27

**Authors:** James M. Parkhurst, Maud Dumoux, Mark Basham, Daniel Clare, C. Alistair Siebert, Trond Varslot, Angus Kirkland, James H. Naismith, Gwyndaf Evans

**Affiliations:** ^1^ Rosalind Franklin Institute, Harwell Science and Innovation Campus, Didcot OX11 0FA, UK; ^2^ Diamond Light Source, Harwell Science and Innovation Campus, Didcot OX11 0DE, UK; ^3^ Electron Physical Science Imaging Centre, Diamond Light Source, Harwell Science and Innovation Campus, Didcot OX11 0DE, UK; ^4^ Thermo Fisher Scientific, Vlastimila Pecha, Brno, Czech Republic; ^5^ Department of Materials, University of Oxford, Parks Road, Oxford OX1 3PH, UK; ^6^ Division of Structural Biology, University of Oxford, Roosevelt Drive, Oxford OX3 7BN, UK

**Keywords:** digital twin, electron microscopy, tomography, multislice simulation

## Abstract

In cryo-electron tomography (cryo-ET) of biological samples, the quality of tomographic reconstructions can vary depending on the transmission electron microscope (TEM) instrument and data acquisition parameters. In this paper, we present Parakeet, a ‘digital twin’ software pipeline for the assessment of the impact of various TEM experiment parameters on the quality of three-dimensional tomographic reconstructions. The Parakeet digital twin is a digital model that can be used to optimize the performance and utilization of a physical instrument to enable *in silico* optimization of sample geometries, data acquisition schemes and instrument parameters. The digital twin performs virtual sample generation, TEM image simulation, and tilt series reconstruction and analysis within a convenient software framework. As well as being able to produce physically realistic simulated cryo-ET datasets to aid the development of tomographic reconstruction and subtomogram averaging programs, Parakeet aims to enable convenient assessment of the effects of different microscope parameters and data acquisition parameters on reconstruction quality. To illustrate the use of the software, we present the example of a quantitative analysis of missing wedge artefacts on simulated planar and cylindrical biological samples and discuss how data collection parameters can be modified for cylindrical samples where a full 180° tilt range might be measured.

## Graphical abstract

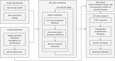

## 1. Introduction

In cryo-electron tomography (cryo-ET), a series of projection images of a sample obtained using a transmission electron microscope (TEM) are recorded at different angles through the sample. From the Fourier projection slice theorem [1,2], a single projection image in real space is equivalent to a single slice through Fourier space. Therefore, by filling three-dimensional Fourier space using projections from many angles, a tomographic reconstruction can be recovered. The quality of the tomographic reconstructions in cryo-ET of biological samples can vary greatly depending on the TEM instrument specifications and experimental data acquisition parameters used; consequently, a significant amount of development time is dedicated to increasing the quality and efficiency of imaging acquisition [3–6], improving data acquisition software [7], and refining data analysis and reconstruction software [8]. For this reason, it can be helpful for developers to first implement new ideas and proposed developments within a ‘digital twin’ of the TEM to enable offline optimization of sample characteristics, data acquisition schemes and instrument parameters. For example, in a digital twin of the TEM, it is trivial to vary sample characteristics (such as the sample thickness, composition and sensitivity to beam damage), instrument parameters (such as electron energy, energy spread, microscope aberrations, and the presence of a phase plate) and data acquisition parameters (such as electron dose and sample tilt range). While some of these experimental parameters may also be easily varied in a physical experiment, modifying or controlling for others may be somewhat more challenging or time consuming.

In its most general form, a digital twin is a digital model representing a physical object that can be used to optimize the performance and utilization of the physical object. In the specific case of cryo-ET, a digital twin can be used to determine optimal data acquisition schemes and instrument parameters and provide phantom test datasets that can be used in the development of data processing software. This is increasingly important when the instrument is of a new design and representative data is not available from a real instrument. First the digital twin must implement a physically realistic model of the sample used in the TEM. In the case of cryo-ET of biological specimens, this sample may be represented by a collection of biological macromolecules embedded in a large volume of amorphous ice [9], typically with a planar geometry. Next, the digital twin must be able to accurately generate physically realistic TEM images of the sample and offer the same range of instrument and data acquisition parameter values. In this case, TEM simulations are a key component in the creation of the digital twin modelling a physically realistic microscope, sample and detector. In EM, the physics of image formation are well understood, and realistic images are routinely simulated using the multislice algorithm [10,11]. As well as assisting in the interpretation of experimentally acquired cryo-EM images, simulations have been used to provide insight into possible experimental limitations of different data acquisition schemes by performing *in silico* experiments that sample the available space of data acquisition parameters. For example, Zhang *et al.* [12] used simulated EM images with an ideal phase plate to show that the structure of hen egg white lysozyme could in principle be solved via cryo-electron microscopy (cryo-EM), and Palmer & Löwe [13] used simulations to assess the effects of missing wedge artefacts in cryo-ET reconstructions.

Finally, in order to use the simulated EM images to evaluate the effects of varying different instrument parameters, the digital twin must be able to produce three-dimensional reconstructions of the object of interest from the simulated images using standard algorithms. The digital twin must then have the ability to compare the reconstructed object with the reference object to determine the overall quality of reconstruction. In cryo-ET, the reconstruction quality is most commonly assessed using the Fourier shell correlation (FSC) [14]. Comparing the reconstructed volume, derived from the simulated images, with the original reference model ‘closes the loop’ and enables optimization of sample preparation, instrument and data acquisition parameters for the physical instrument.

In this paper, we present a digital twin software pipeline, Parakeet (**P**rogram for **A**nalysis and **R**econstruction of **A**rtificial data for **K**ryo **E**l**E**ctron **T**omography), to analyse the impact of varying data acquisition and analysis parameters on reconstruction quality for cryo-ET. A fundamental problem in tomography is the missing wedge resulting from the use of a limited range of tilt angles. Due to physical constraints, such as the limited space between the objective lens pole pieces and the dimensions of the specimen holder [15], tilt stages in current generations of electron microscopes tend to have a limited rotation range of ±70° [16–18]; indeed, in practice, cryo-ET datasets are collected with a tilt range of around ±60° [19]. New sample preparation, hardware and software developments in the field, such as the use of cylindrical specimens [13], dual-axis data acquisition schemes [4,20–23] and advanced reconstruction algorithms [24–28], have focussed on trying to alleviate this problem. To illustrate the use of Parakeet, we apply it to the problem of quantifying the impact of the missing wedge on reconstruction quality.

## 2. Digital twin

### 2.1. Design overview

**Figure 1.**
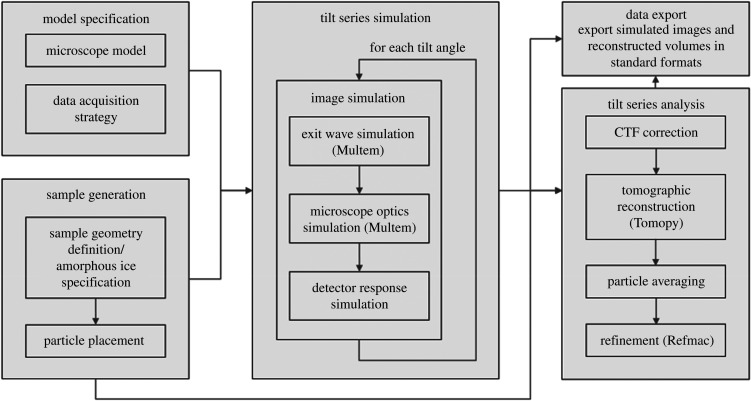
Flow chart describing the Parakeet digital twin software pipeline.

The core aim of Parakeet is to enable convenient assessment of the effects of varying data acquisition and analysis parameters on the quality of tomographic reconstructions of biological samples. The workflow in the digital twin can be decomposed into discrete tasks that exchange information through data files as shown in figure 1. The first step is to describe the microscope model and the desired data acquisition strategy. A virtual sample is then generated with the desired geometry which contains a given number of particles with either specified or random positions and orientations embedded in amorphous ice. To simulate a tilt series of images, multislice TEM simulations [10,11] of biological macromolecules are performed. These simulations model the propagation and scattering of an electron wave through the representation of the atomic model of a specimen. The microscope optics and detector response are then introduced to yield the final simulated image. By rotating the virtual sample, a tilt series can be simulated from the atomic model. Once a simulated tilt series has been acquired, the data analysis component of the digital twin is used to apply a CTF correction to each of the projections in the tilt series. Finally, the projections are reconstructed using standard tomographic reconstruction algorithms and, to ‘close the loop’, the reconstructions are compared with the original known reference atomic model, providing an objective and quantitative assessment of the quality of reconstruction under different simulated data collection strategies. The specimen model is stored using HDF5 (https://www.hdfgroup.org/hdf5), and the simulated images can be exported into either HDF5 format or MRC format [29] using the mrcfile python library [30] with the correct FEI extended header information present in order to provide metadata compatible with other data analysis software. Therefore, Parakeet can be used to provide simulated test data that can be readily imported into external software programs to enable, for example, optimization of tomographic reconstruction and subtomogram averaging (STA) software. Reconstructed volumes are exported in MRC format. The software is open source, written in a combination of C++ and Python, and can be obtained online from the Rosalind Franklin Institute GitHub repository [31].

### 2.2. Model specification

The microscope model is built from a set of models representing the beam, detector and objective lens, the data acquisition scheme is represented by a scan model, and the specimen is represented by a sample model. These models are specified via a YAML (https://yaml.org/) configuration file which provides a convenient metadata serialization format that is both human readable and supported by a wide variety of programming languages.

— *Beam model.* This configures the beam energy, energy spread, source spread and electron flux. It also allows the beam drift to be configured as a function of tilt angle using either a random or sinusoidal model.
— *Detector model.* This configures the number and size of the pixels and configures the detective quantum efficiency (DQE) model.
— *Lens model.* This specifies the objective lens aberrations such as the defocus, spherical aberration and chromatic aberration. It also allows a phase plate to be configured *via* an arbitrary phase shift.
— *Scan model.* This specifies the axis of rotation, the exposure time for each image, the number of images, and the angle and translation between each image in the tilt series.
— *Sample model.* This specifies the geometry of the sample which can be either a plane or a cylinder, configures the amorphous ice model and defines the number, type, positions and orientations of particles in the sample.

### 2.3. Sample generation

The Protein Data Bank (PDB) [32] contains a repository of thousands of biological macromolecules which can be readily accessed to act as a foundation for building a digital twin of the sample. After first defining the overall shape and size of the sample—either planar or cylindrical—the number and type of particles (given by their PDB IDs) to include in the sample volume are then specified. If a single particle is specified, then by default the particle is positioned in the centre of the sample volume and field of view. If multiple particles are specified, they can either be positioned with predefined positions and orientations (provided these positions do not overlap), or they can be assigned random positions and orientations within the sample volume. Particles are only allowed within the defined sample volume and are not allowed to overlap with the surface of the sample volume. Once the sample volume is defined, the atomic model (which may be composed of many particles of different types and orientations) can be ‘milled’ into arbitrary shapes by removing atoms outside defined regions.

**Figure 2.**
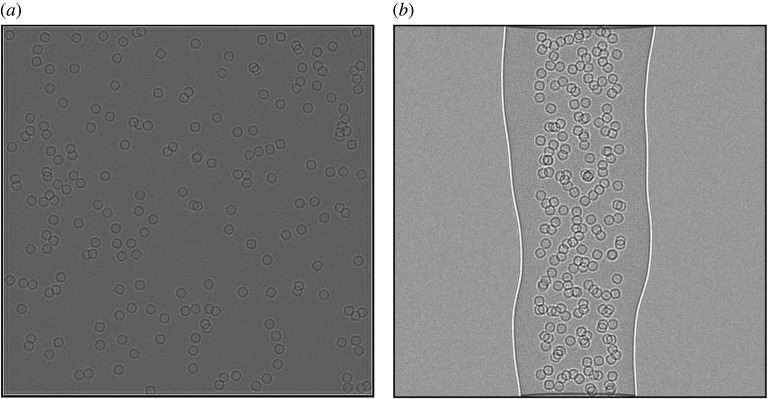
(*a*) A large planar lamella sample of size 4000 Å × 4000 Å × 1500 Å containing 200 apoferritin particles. (*b*) A cylindrical volume with diameter 1500 Å with ‘natural’ edges containing 200 apoferritin particles.

An important aspect of the atomic model of the sample is the amorphous ice component, which by volume may account for a substantial portion of the atoms in the model depending on the thickness of the sample. The digital twin implements two methods for the generation of the amorphous ice within the sample. The first is to simply generate water molecules with random positions and orientations within the sample volume. In this model, water molecules may be placed at unphysical distances relative to one another. An alternative, improved fully atomic model of the amorphous ice, would require molecular dynamics simulations to relax the water molecules to physically realistic distances; however, this is very computationally intensive for the sample volumes considered here and is not discussed. The digital twin also implements a continuum approach using a Gaussian random field (GRF) model for the atomic potential of the amorphous ice in which the ice component is modelled as Fourier filtered noise with a given power spectrum to match the expected correlations for a physically realistic ice model. The GRF approach is more computationally efficient than the atom-based approach. Figure 2 shows a planar and cylindrical sample generated using the GRF approach. The model can also be defined to have ‘natural’ shapes which deviate from the ideal shape of a plane or cylinder. For example, in the case of the cylinder, the radius is parameterized along the length of the cylinder to allow different radii and offsets which are then interpolated using cubic splines.

### 2.4. Tilt series simulation

In order to simulate the TEM images, Parakeet uses the MULTEM library [33] which provides a GPU accelerated implementation of the multislice algorithm [10,11] and a model for the microscope optics. These algorithms were extended and wrapped using the *Pybind11* C++/Python binding package [34] to create a simple Python API (*python-multem*). The python bindings are open source and can be obtained from the Rosalind Franklin Institute GitHub repository (https://github.com/rosalindfranklininstitute/python-multem).

The multislice algorithm operates by taking the atomic model and dividing the sample into slices of a given thickness along the direction of the electron beam. The thickness of each slice is typically a few angstroms such that each slice can be considered as a weak phase object; in the simulations used here, the slice thickness is 5 Å. Each sample slice is then treated as infinitesimally thin, and for each slice the atomic potential is calculated as the sum of the atomic potentials of the constituent atoms projected onto the infinitesimally thin slice. The calculation for each slice is performed on a discrete grid with a pixel size of 1 Å × 1 Å. The wave function is then transmitted through the slice and propagated to the next slice via Fresnel diffraction. Propagating the wave function through the whole sample gives a complex wave function at the exit surface of the sample.

As described in appendix B, beam damage to the sample is implemented by convolving the electrostatic potential of the specimen with a Gaussian function [12] whose variance, *σ*_B_^2^, can be related to an isotropic *B* factor by *B* = 8*π*^2^*σ_B_*^2^ [35]. This convolution is conveniently performed in Fourier space where the *B* factor filter is expressed as a function of spatial frequency, *q*. The potential from a damaged specimen, *V*, can then be related to the undamaged potential, *V*_0_ by *V* = *F*^−1^[*F*[*V*_0_] exp(−q^2^ *B*/4)]. The isotropic *B* factor is parameterized as a linear function of the total accumulated incident electron dose, *D*_E_, and is given by *B* = 8*π*^2^*D*_E_*S*_E_, where *S*_E_ is the sensitivity coefficient. The value of this coefficient is sample dependent but can be calibrated from the results of X-ray diffraction experiments [36–38], electron diffraction experiments [39] or single-particle cryo-EM experiments [40] and typically takes values between 0.020 and 0.090.

The effects of the microscope optics are modelled by application of a contrast transfer function (CTF), an oscillating complex function of the aberrations in Fourier space, which applies a frequency-dependent phase shift to the exit wave. Spatial and temporal coherence envelopes, which are applicable in the case of the linear imaging approximation, are included which damp the CTF at high resolution. Finally, the detector response, in the form of a frequency-dependent DQE and Poisson counting noise for the number of expected electrons per pixel are added to generate the final simulated images. Typical values for the parameters used in the simulations are summarized in table 1; our choices were guided by what is practical using a Thermo Fisher Scientific Titan Krios instrument [41].

**Table 1.** Typical parameters used in the TEM simulations, chosen to be consistent with parameters for a Thermo Fisher Scientific Titan Krios TEM [41].

parameter	description	value
*E*	energy	300 keV
Δ*f*	defocus	2.5 µm
*C*s	spherical aberration	2.7 mm
*C*c	chromatic aberration	2.7 mm
Δ*I*/*I*	current spread	0.33 ppm
Δ*V*/*V*	voltage spread	0.80 ppm
Δ*E*	energy spread	0.8 eV
*θ*_c_	source spread	0.1 mrad
*d*_px_	pixel size	1 Å
*t*_s_	multislice z-slice thickness	5 Å
	potential approximation	Lobato *et al*. [42]
*S*_E_	beam damage sensitivity coefficient	0.022 A^2^/e^−^

### 2.5. Tilt series analysis

To obtain high-resolution reconstructions, CTF correction [43] or exit wave reconstruction from multiple images [44] must be performed. This is typically done either by phase-flipping, multiplying the Fourier transform of the image by the CTF, or through the use of a Wiener filter [43]. When a single defocus is used, only the contrast inversions can be corrected for. In this case, a Wiener filter may amplify noise at spatial frequencies close to the zero crossings of the CTF and the Fourier components at the zero crossings themselves cannot be corrected at all. In phase-flipping, the contrast inversions are corrected by multiplying the Fourier transform of the image by the sign of the CTF. Phase-flipping has been shown to result in marginally better reconstructions than simply multiplying by the CTF [43] and is therefore implemented in Parakeet.

For two-dimensional single-particle analysis (SPA), where untilted images of thin specimens are used, a single CTF correction can be applied to each image. However, in the case of tomography, when a planar sample is tilted, different parts of the sample will appear at different defoci [45] and this defocus gradient can become significant at high tilt angles. Additionally, for thick samples, different voxels within a sample have different defocus values. In this case, CTF correction using a single defocus value is insufficient and a three-dimensional CTF correction is required. This can be implemented by computing multiple CTF corrected images for each tilt angle and then using the appropriate corrected image pixel for each voxel in the reconstruction according to the location of the voxel within the volume [46]. However, in order to use multiple CTF corrected images per tilt rather than a single image per tilt, this approach requires the core of the standard tomographic reconstruction algorithms to be extended. Alternatively, if the dataset is to be used for STA [47], CTF correction can be applied to reconstruct each particle independently before averaging the individual subtomograms; this is the approach used here, as it permits the use of standard tomographic reconstruction algorithms.

In Parakeet, the CTF corrected tomographic tilt series are reconstructed using a GPU accelerated weighted back projection (WBP) algorithm implemented using the Astra toolbox [48] through the Tomopy python package [49,50]. This is a more robust variant of the filtered back projection algorithm which applies a unique filter to each projection based on the total number of projections and their rotational distribution, rather than applying the same ramp filter to all projections [51,52]. Perhaps the most widely used of the alternatives to the back projection family of algorithms is the simultaneous iterative reconstruction technique (SIRT) algorithm which has been shown to give better-quality reconstructions than WBP in the presence of missing and low SNR data (both features of cryo-ET) [25,53,54]. However, SIRT requires careful selection of the number of iterations; although each iteration increases the agreement between the forward projection of the reconstruction with the observed images, this comes with a concomitant increase in noise features and thus a compromise must be found [25]. Other advanced algorithms using expectation maximization [55] and other iterative approaches [56] have also been reported. The WBP algorithm is implemented here since it is used in many cryo-ET reconstructions and requires no additional parameter optimization for each tilt series, making it appropriate for use in an automated pipeline.

Tomopy is used because it gives access to various reconstruction algorithms that may not be implemented in standard cryo-ET packages; it therefore allows greater flexibility in evaluating different reconstruction strategies. Additionally, since the package is written in Python, it can be seamlessly incorporated into data processing scripts for automation and analysis in a way that is more difficult for packages lacking an accessible API [57]. Finally, individual particle reconstructions are manually picked from the reconstructed tomograms using the known simulated particle positions and orientations; the final reconstruction is obtained by taking the Fourier transforms of the individual particle reconstructions and averaging them while applying a missing wedge mask to each particle in Fourier space.

As the images are simulated from a known atomic model, it is straightforward to fit the original atomic model back onto the reconstructed map. In order to do this, refinement of the atomic model is performed using REFMAC5 [58] with rigid body restraints. By measuring the fit of model to map, REFMAC5 provides an objective analysis of the quality of different reconstructions. We use the FSC average [14,59] which is defined by

$${\mathrm{FSC}}_{\mathrm{average}}=\frac{\sum_{i=1}^{N_{\mathrm{shell}}}N_{i}{\mathrm{FSC}}_{i}}{\sum_{i=1}^{N_{\mathrm{shell}}}N_{i}},$$

where *N_i_* is the number of elements in a shell and FSC*_i_* is the FSC within a shell, *i*.

## 3. Application example

The missing wedge problem has been the topic of numerous studies and efforts aimed at minimizing or correcting its deleterious effects on the quality of the tomographic reconstruction [13,60–62]. Here, Parakeet is used to quantify the impact of the missing wedge with reference to data without a missing wedge and consider the hypothetical scenario of using a cylindrical sample with a tilt stage that offers full rotation capability.

### 3.1. The missing wedge problem

The missing wedge problem is used as an example because it is one of the most pressing issues in the field of cryo-ET with complex data collection strategies and new sample preparation, hardware and software developments focused on alleviating it. In order to avoid artefacts relating to missing data, when reconstructing a three-dimensional object, the amplitudes across all spatial frequencies (to the desired resolution) need to be sampled. For objects with no symmetry, this requires a set of projections to be acquired over a full 180° rotation range. Where fewer data are recorded, spatial frequencies in some regions of Fourier space will be absent and the real space reconstruction will contain artefacts. In other words, the missing wedge problem arises when an entire region of Fourier space has not been sampled due to the use of a restricted rotation range. The missing wedge problem results in the introduction of artefacts, such as halos and streaking around objects with high contrast [13], anisotropic resolution in the reconstructed volume [16,63] and an incomplete three-dimensional representation of the specimen [47].

**Figure 3.**
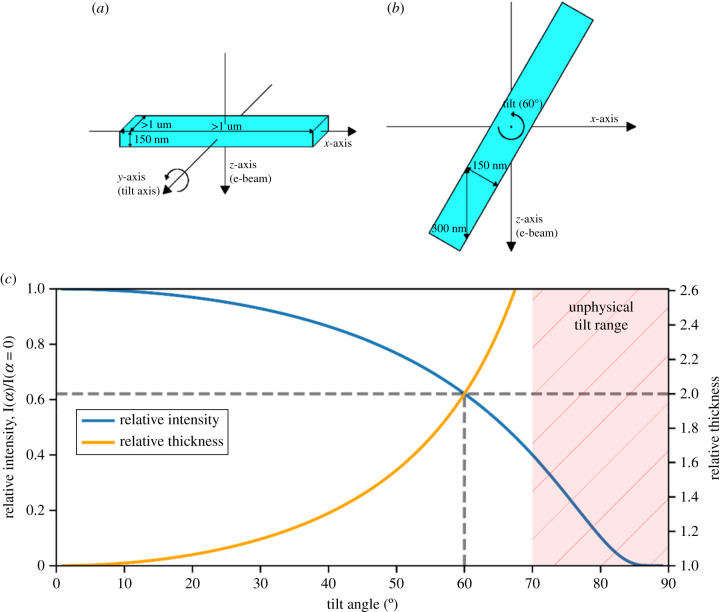
The lamella geometry. (*a*) Coordinate system and typical sizes of the lamella along with each axis at zero tilt. (*b*) Lamella tilted to 60° with an effective thickness along with the beam direction of twice the true thickness. (*c*) Relative thickness (orange, right axis) increasing for a planar sample at high tilt angle as *D*/*D*_0_ = 1/cos(*α*); the relative intensity (blue, left axis) as a function of tilt angle follows *I*/*I*_0_ = exp((1 − 1/cos(*α*)) *D*_0_/*λ*), plotted here for *D*_0_ = 150 nm and *λ* = 314 nm [39]. The unphysical tilt region (for a lamella) is shown by the red hatched area. Relative thickness and intensity at a tilt angle of 60° are shown by the dashed lines.

In the typical cryo-ET experiment, frozen-hydrated samples are prepared as planar lamellae which are ‘thin’ along the incident beam direction (at zero tilt) and ‘thick’ orthogonal to this [19], as shown in figure 3. Typically, cryo-ET datasets are collected with a tilt range of around ±60° [19], corresponding to a missing wedge of 60°. However, planar lamellae introduce additional complications because, as the planar lamella is tilted, its effective thickness along with the beam direction increases at high tilt angles resulting in chromatic blurring due to the increased inelastic scattering [60]. An energy filter can be used to increase the signal to noise by removing the inelastically scattered electrons from the image; however, this results in a reduction in the intensity of the overall signal which becomes increasingly severe as more electrons are scattered inelastically and no longer contribute to the signal. A proposed solution to this problem is to use cylindrical samples which allow a full 180° tilt range. Such samples have been used in soft X-ray tomography [62], and in EM in materials science [60], with reconstructions showing improved reconstruction quality and isotropic resolution [61]. Palmer & Löwe [13] describe the use of a cylindrical specimen holder for cryo-ET of biological samples; however, the preparation of such samples has so far proved to be difficult and in order to become a useful technique, problems associated with the sample preparation must be addressed. For large cellular samples, high-pressure freezing can be used in the sample preparation to ensure vitrification [64,65] and a promising approach would be to then use a focussed ion beam to ‘mill’ these samples to achieve the desired cylindrical geometry [66]; this has already been demonstrated for radiation-resistant samples [23,26,61,67,68]. Once this becomes routinely possible, it is likely that on-axis tomography with cylindrical samples will quickly become the method of choice for high-resolution cryo-ET data acquisition. Therefore, it is instructive to use the digital twin to determine what gains can be obtained in the quality of reconstruction for biological macromolecules using cylindrical samples rather than planar samples in the absence of a missing wedge artefact in order to provide motivation for the development of new advanced sample preparation techniques.

### 3.2. Simulated data

For the simulations reported here, a model of apoferritin and a ribosome available from the PDB [32] were used. The PDB entry for apoferritin, 6Z6U [69], is resolved to 1.25 Å and was determined by cryo-EM SPA. It has a total structural weight of 511.09 kDa and 38 846 atoms in the model. The PDB entry for the ribosome, 4V5D [70], is resolved to 3.50 Å and was determined by X-ray diffraction. It has a total structural weight of 4516.21 kDa and 296 042 atoms in the model.

In general, for single-particle samples, given the time-consuming sample preparation and data acquisition required for tomography, it is often more convenient to use standard SPA approaches rather than tomography. Cryo-ET with cylindrical samples is hence likely to provide the greatest benefit to the analysis of more complex cellular samples. However, single-particle samples are convenient for the purpose of evaluating different data acquisition methodologies in cryo-ET, both experimentally and through simulation, since they provide a straightforward method to assess the quality of reconstructions from different schemes through the correlation between the reconstruction and the known structure. Apoferritin was chosen because it has a high degree of symmetry which allows the quality of the reconstructions along with different directions to be compared. Second, its alpha helices can be identified even in low-resolution maps which aids in the qualitative assessment of the reconstruction quality. Apoferritin is also commonly used as a test sample for cryo-EM applications [71]. Ribosome was used because it has characteristics complementary to apoferritin in that it is a larger structure with no structural symmetry

**Figure 4.**
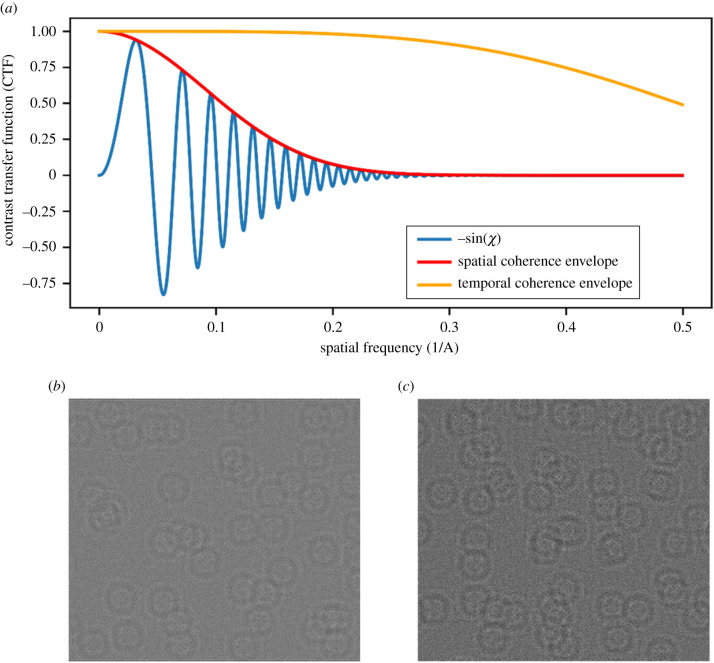
(*a*) Imaginary component of the CTF using the parameters in table 1. (*b*,*c*) Cropped 100 × 100 nm sections of the zero-tilt image for (*b*) a planar sample and (*c*) a cylinder sample containing apoferritin molecules where every particle has a random position and the same known preferred orientation.

The TEM parameters used in the simulations are shown in table 1. The imaginary component of the CTF given these parameters, including the spatial and temporal coherence envelopes, is shown in figure 4 along with a cropped 100 × 100 nm section of a simulated image for both the planar and cylindrical samples. The simulated planar samples had *x*-widths of 1000 nm and *z*-depths of 100 nm, 150 nm and 200 nm. The simulated cylindrical samples had diameters of 100 nm, 150 nm and 200 nm. Each samples contained 15 k randomly positioned molecules each with the same pre-determined orientation; for the apoferritin simulations, the particles were oriented such that a symmetry axis was aligned with the sample to highlight the effect of the missing wedge on reconstructions. Although having only one orientation is not necessarily representative of all physical samples, some of which may have a preferred particle orientation but most of which will include particles with a variety of orientations, the inclusion of random orientations would effectively reduce the effects of the missing wedge on the reconstructions. The simulated projections have an isotropic pixel size of 1 Å.

### 3.3. Variation of reconstruction quality for different simulated data collection regimes

**Figure 5.**
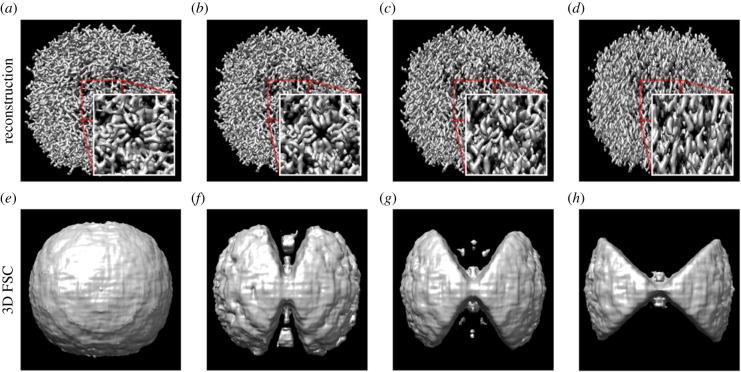
Reconstructions of a simulated apoferritin molecule from tilt series with a maximum tilt angle of (*a*) ± 90°, (*b*) ± 75°, (*c*) ± 60° and (*d*) ± 45°. The features along with the *z*-axis are progressively blurred as the size of the missing wedge increases. The local 3D FSC for the reconstruction with the known structure at a FSC threshold level of 0.85 for (*e*) ± 90°, (*f*) ± 75°, (*g*) ± 60° and (*h*) ± 45°.

Apoferritin reconstructions were performed using particles embedded within a cylindrical sample with no missing wedge and missing wedges of 30°, 60° and 90° (figure 5). As expected, the quality of the reconstruction decreases as the missing wedge increases and most notably features along with the z-axis are blurred. The local three-dimensional FSC shows that the correlation between the true map and reconstructed map also decreases with an increasing missing wedge, giving an objective and quantitative assessment of the extent of degradation in the reconstruction.

Keeping the total simulated electron dose constant, we analysed planar and cylindrical samples with 90 projections per tilt series and separately with fixed 2° tilt increments. The data were simulated and analysed for a range of maximum tilt angles from 30° to 90° for sample thicknesses of 100 nm, 150 nm and 200 nm. In each case, the simulated data acquisition was performed using a standard dose symmetric data acquisition scheme where the projections were acquired in order of absolute tilt angle with projections at low tilt angles being acquired first [3]. Keeping the number of projections constant over different tilt ranges allows the tilt increment to vary. In the second approach, the tilt increment is kept constant, allowing the number of projections to vary depending on the overall range of tilt angles. Hence these experiments interrogate which of two differential samplings of the three-dimensional object provides the highest quality reconstruction as assessed using the FSC average, FSC in high- and low-resolution bins and anisotropic FSC average in the three reciprocal space planes.

**Figure 6.**
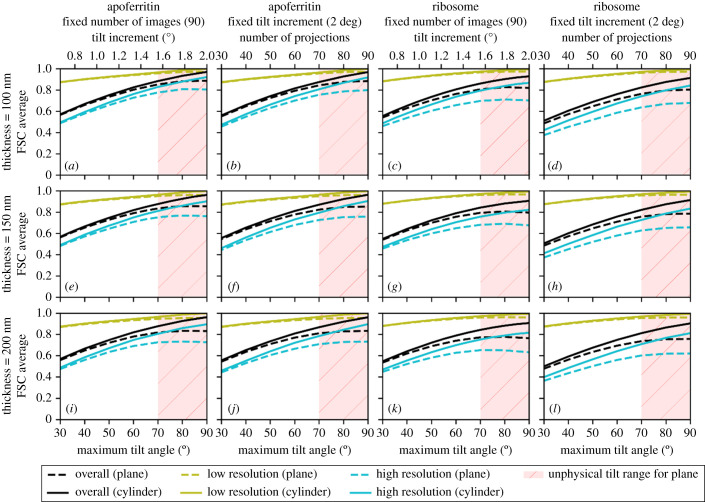
The effect of increasing the maximum tilt angle on the quality of reconstructed data for planar samples (dashed lines) and cylindrical samples (solid lines), for apoferritin (*a*,*b*,*e*,*f*,*i*,*j*) and ribosome samples (*c*,*d*,*g*,*h*,*k*,*l*), with a fixed number of projections and a fixed tilt increment, for sample, thicknesses of 100 nm (*a*–*d*), 150 nm (*e*–*h*) and 200 nm (*i*–*l*). The quality of the reconstruction is assessed by the overall FSC average and by the FSC in high- and low-resolution bins. As the maximum tilt angle increases, the quality of the reconstruction generally improves in each case. For the planar samples, high maximum tilt angles are considered to be unphysical due to sample and mechanical limitations and are shaded in red. In all cases, the cylindrical samples show marginally better reconstruction quality than the planar sample.

**Figure 7.**
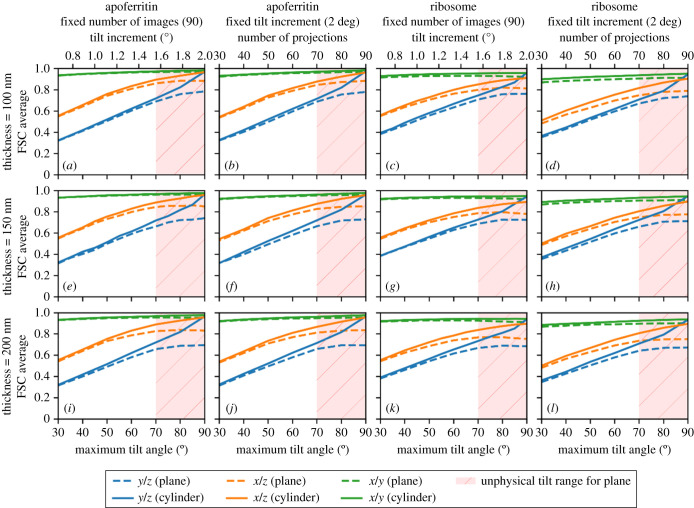
The effect of increasing the maximum tilt angle on the anisotropic FSC average of reconstructed data in the reciprocal *x/y*, *x/z* and *y/z* planes for planar samples (dashed lines) and cylindrical samples (solid lines), for apoferritin (*a*,*b*,*e*,*f*,*i*,*j*) ) and ribosome samples (*c*,*d*,*g*,*h*,*k*,*l*), with a fixed number of projections and a fixed tilt increment, for sample thicknesses of 100 nm (*a*–*d*), 150 nm (*e*–*h*) and 200 nm (*i*–*l*). For the planar samples, high maximum tilt angles are considered to be unphysical due to sample and mechanical limitations and are shaded in red.

The FSC average and FSC in high- and low-resolution bins were plotted as a function of the maximum tilt angle for the planar and cylindrical apoferritin and ribosome samples for a constant number of projections and constant tilt increment (figure 6). In addition to the overall FSC average, the FSC average along the *x/y*, *x/z* and *y/z* planes (figure 7) was evaluated in order to probe the anisotropic effect of the missing wedge artefacts. As expected, the quality of the reconstruction, as assessed by the FSC average, improves as the maximum tilt angle increases. However, for a planar sample, images at a high tilt angle would be expected to have poorer SNR than images at a low tilt angle due to the increased projected thickness at a high tilt angle. With poorer SNR, the images taken at high tilt angles will contribute less information thus amplifying the missing wedge problem [13]. We observed that the quality of the reconstructions for planar samples did not increase beyond a maximum tilt angle of around 70°, corresponding to the typical physical limits of most instruments. For a planar sample, over the range of sample thicknesses considered here, at a 70° tilt, the deleterious effect of the increased apparent sample thickness (figure 1) outweighed the benefit of reducing the missing wedge. Our simulations in fact understate the problem, since the effects of the sample holder in a physical experiment will further degrade very high tilt measurements by shadowing.

**Figure 8.**
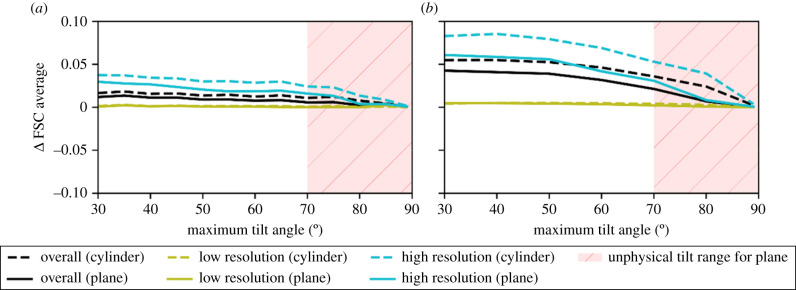
The difference in FSC average between the fixed number of projections and fixed tilt increment data acquisition schemes as a function of the maximum tilt angle for planar samples (dashed lines) and cylindrical samples (solid lines), for apoferritin (*a*) and ribosome samples (*b*). The plots show the overall FSC average and the FSC in high- and low-resolution bins. For the planar samples, high maximum tilt angles are considered to be unphysical due to sample and mechanical limitations and are shaded in red. In each case, the data acquisition scheme with a fixed number of images shows better reconstruction quality than the scheme with a fixed tilt increment.

As expected for the cylindrical sample, with a constant apparent thickness as a function of rotation, the quality of the reconstruction increases as the maximum tilt angle is increased to 90° and as expected outperforms the planar sample. For the cylindrical sample, the FSC average in the reciprocal *x/y* plane is relatively insensitive to the size of the missing wedge; however, the reciprocal *x/z* and reciprocal *y/z* planes are very sensitive to the extent of the missing wedge. Even at more limited tilt angles, the cylindrical sample gives a slightly higher quality reconstruction. Tilt series with a fixed number of projections for a given tilt range produced marginally better-quality reconstructions than the equivalent tilt series with the same maximum tilt angle and a fixed tilt increment of 2° (figure 8). This is to be expected since the number of projections is closely related to the quality of the reconstructions and reducing the number of projections as well as the range of tilt angles will reduce the quality of the reconstruction even further. The difference in reconstruction quality is especially evident at high resolution.

### 3.4. Variation of reconstruction quality with number of projections

**Figure 9.**
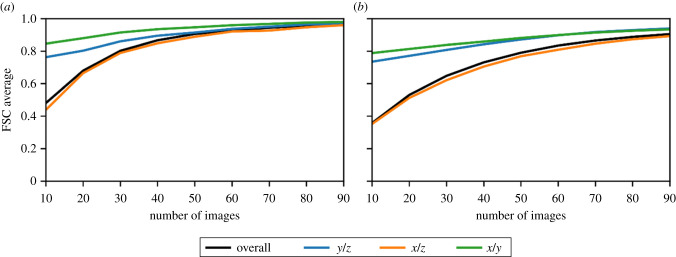
The quality of the reconstruction as a function of number of projections for a cylindrical specimen with no missing wedge. As the number of projections increases, the quality of the reconstruction increases until it begins to plateau. The overall FSC average is shown in black with the FSC average in the reciprocal *x/y*, *x/z* and *y/z* planes also shown.

The reconstruction quality, as assessed by the FSC average, of the 180° dataset with an increasing number of projections was evaluated for the 150 nm thick cylindrical apoferritin and ribosome samples assuming perfect image alignment as shown in figure 9. As the number of projections is increased, the quality of the reconstruction improves to a maximum value which then remains approximately constant as other factors affecting the reconstruction quality, such as the discrete sampling of the detector, begin to dominate over the rotational sampling of the volume. It is well known that the achievable resolution of a tomographic reconstruction is given by the Crowther criterion [2] which, ignoring any symmetry considerations or potential gains from averaging, is given by *d* = π D/N, where *d* is the achievable resolution, *D* is the diameter of the sample and *N* is the number of projections. This equation assumes that the resolution is not limited by the Nyquist sampling frequency of the detector, the sample is spherical with no additional symmetry, and that *N* projections completely fill Fourier space. In practice, when a tilt range (−α, *α*) is used, and *α* < π/2, the resolution is anisotropic such that, typically, *d*_y_ < *d*_x_ < *d*_z_ [26] as previously illustrated in figure 7.

**Figure 10.**
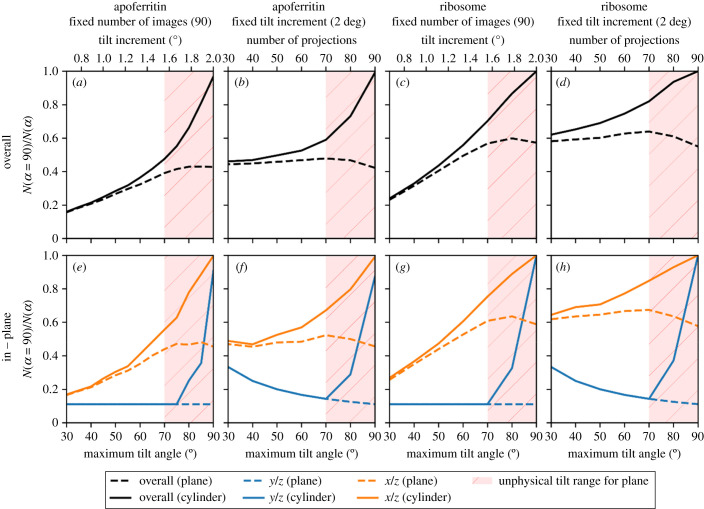
The fraction of projections required for a cylindrical sample with no missing wedge to achieve the same quality of reconstruction as the missing wedge datasets for a planar sample (dashed lines) and cylindrical sample (solid lines), for apoferritin (*a*,*b*,*e*,*f*) and ribosomes (*c*,*d*,*g*,*h*), with a fixed number of projections and a fixed tilt increment using the FSC average overall (*a*–*d*) and in the reciprocal *x/z* and *y/z* planes (*e*–*h*). For the planar samples, high maximum tilt angles considered to be unphysical due to sample and mechanical limitations are shaded in red.

We have explored whether, given no missing wedge, fewer projections are needed to give the same reconstruction quality as data with a missing wedge. As shown in figure 10, for the 150 nm thick cylindrical apoferritin and ribosome samples, the number of projections required to achieve the same reconstruction quality is reduced by more than half when compared to corresponding planar samples with a 120° tilt range. This effect is approximately linear until a maximum rotation of around 70° where the increased apparent thickness of the planar sample at high tilt angles begins to reduce the benefit of including higher tilt angle data. The effect is even more pronounced in the reciprocal *y/z* plane where the reduction is 10-fold. This is highly beneficial since the limited dose tolerance of a biological sample needs to be fractionated over the entire tilt series and hence a larger number of projections means a lower SNR per projection. The SNR is critical for alignment of the tilt series before reconstruction, thus although increasing the number of projections increases the quality of reconstruction, the gain may be offset by errors in alignment. When fewer projections are needed to obtain the same quality of reconstruction, these projections can individually have higher SNR and thus reduced alignment errors.

## 4. Conclusion

Cryo-ET is revolutionizing cell biology, and there is significant interest in improving the technique. Current experimental approaches suffer from a missing wedge of data. Since the potential for the redesign of hardware is limited, it is important to quantify the gains of any redesign. We report a digital twin software pipeline, Parakeet, which is capable of simulating tomographic tilt series of TEM projection images, reconstructing and analysing them. This has allowed us to quantify the effects of the missing wedge and sample geometry. For a planar lamella, the best reconstruction quality for a fixed number of projections was obtained with a maximum tilt angle of less than 90° since projections at higher tilt angles contributed little to the reconstruction and added noise. For a cylindrical sample, however, the quality of the reconstruction increased as the tilt range approached the full hemisphere. In addition, a cylindrical sample with the same thickness as the planar sample was shown to require fewer projections, spaced over the hemisphere, to achieve the same quality of reconstruction as a planar sample with a missing wedge. The software is open source and available to download from the Rosalind Franklin Institute GitHub page [31].

## Data accessibility

1. The atomic models of the apoferritin and ribosome used are publicly available and can be downloaded from the Protein Data Bank. 2. All the software described is free and open source and can be downloaded from the GitHub repository at https://github.com/rosalindfranklininstitute/amplus-digital-twin.

## Appendix A. Command line programs

The main command line programs that constitute the Parakeet software pipeline are shown below. These command line programs roughly correspond to the steps of the flow chart in figure 2.

— *parakeet.config.show*
  This program is used to generate a new YAML configuration file (config.yaml) defining the microscope model and data acquisition strategy. This config file can then be used in all the subsequent command line steps. However, before proceeding, the config file may need to be manually modified by the user to customize some aspects of the simulation.
— *parakeet.sample.new* [-c config.yaml]
  This program initializes the sample with the desired geometry and amorphous ice specification. The program takes a configuration file (by default assumed to be named config.yaml) and generates a skeleton HDF5 file (by default named sample.h5). The sample geometry can either be cuboid or cylindrical; the size of the cuboid or cylinder and its location and orientation with respect to the beam are specified here.
— *parakeet.sample.add_molecules* [-c config.yaml]
  This program adds a number of particles to the sample atomic model with either given or random position and orientation. The program used the same configuration file and takes as input the sample HDF5 file generated by the *parakeet.sample.new* program which is modified in place (i.e. multiple invocations of the command will modify the same file rather than overwriting the results of the previous function call).
— *parakeet.simulate.exit_wave* [-c config.yaml]
  This program uses the multislice algorithm to simulate the exit wave of the electron through the sample. The program takes as input the config file and the sample HDF5 file generated in the previous steps. The exit wave is written out to either a HDF5 file (by default named exit_wave.h5) or an MRC file containing the simulated images for the whole tilt series.
— *parakeet.simulate.optics* [-c config.yaml]
  This program simulates the effects of the microscope optics *via* the CTF. The program takes as input the config file and the exit wave file generated by the *parakeet.simulate.exit_wave* program. The tilt series with the microscope optics applied is then written out to either a HDF5 file (by default named optics.h5) or an MRC file containing the simulated images for the whole tilt series.
— *parakeet.simulate.image* [-c config.yaml]
  This program simulates the detector response by adding the effect of the DQE and applying Poisson noise. The program takes as input the config file and the optics file generated by the *parakeet.simulate.optics* program. The tilt series images are then written out to either a HDF5 file (by default named image.h5) or an MRC file containing the simulated images for the whole tilt series.
— *parakeet.analyse.reconstruct* [-c config.yaml]
  This program performs CTF correction and reconstructs the simulated tilt series. The program takes as input the same config file as used throughout the simulation process and the tilt images in MRC format. After reconstructing the tomogram, the program outputs an MRC file (by default named rec.mrc).
— *parakeet.analyse.average_particles* [-c config.yaml]
  This program uses the given particle positions and orientations and averages the particle reconstructions. The program takes as input the configuration file, the sample HDF5 file and the reconstruction file output from the *parakeet.analyse.reconstruct* program. The particle reconstructions are extracted using the given particle positions and orientations and averaged in two halves. The program outputs these two half reconstructions in two files by default named half1.mrc and half2.mrc.

## Appendix B. Beam damage model

The first-order effects of beam damage to the sample can be implemented by convolving the electrostatic potential with a Gaussian function [12] whose variance, *σ*_B_^2^, can be related to an isotropic *B* factor by *B* = 8*π*^2^*σ_B_*^2^ [35]. This convolution is most conveniently performed in Fourier space where the *B* factor filter is expressed as a function of spatial frequency, *q*. The potential from a damaged specimen, *V*, can then be given in terms of the undamaged potential, *V*_0_, by

$$V=F^{-1}[F[V_{0}]e^{-q^{2}B/4}].$$

The isotropic *B* factor for protein crystals has been shown to vary linearly with the absorbed X-ray dose [36,37]. This dependence can be encapsulated in a coefficient of sensitivity to absorbed dose which gives the relative change in the isotropic *B* factor to the change in dose [37]:

$$S_{AD}=\frac{\Delta B}{8\pi^{2}\Delta D}.$$

This coefficient, which gives the slope of the linear relationship between the isotropic *B* factor and dose, is thought to take similar values for most protein crystals [37,72]. A typical value for this coefficient for cryo-cooled crystals at a temperature of 100 K is *S*_AD_ = 0.012 A^2^ MGy^−1^ [38]. The isotropic *B* factor for a given absorbed dose is then

$$B=8\pi^{2}D_{MGy}S_{AD}.$$

In Parakeet, rather than the sensitivity to absorbed dose in MGy, the sensitivity to the incident dose in e^−^/A^2^ is required. The dose in *G*y for an incident number of electrons can be defined as follows, where *D*_e_ is the dose in electrons per unit area, *ρ* is the specimen density (kg m^−3^), *E*_m_ is the mean energy loss (J), and *λ*_i_ is the total mean free path (m) for the inelastic scattering of electrons [73]:

$$D_{Gy}=\frac{D_{e}E_{m}}{\rho \lambda_{i}}.$$

The inelastic mean free path is given by *λ*_i_ = 1/(*Nσ*_i_), where *N* = *ρ*N_A_/*A* is the number of particles per unit area given in terms of the sample density, *ρ*, Avogadro's number, *N*_A_ and the atomic mass, *A*; and *σ*_i_ is the total inelastic scattering cross-section which can be written in terms of the atomic number, *Z*, the velocity of the electrons, *β*, the electron acceleration voltage, *V*_0_, and the rest energy of the electron, *mc*^2^, as follows [74]:

$$\sigma_{i}=\frac{1.5\times 10^{-6}Z^{1/2}}{\beta^{2}}\;ln\;\left[2/\left(\frac{E_{m}}{\beta^{2}(V_{0}+mc^{2})}\right)\right]{\mathrm{nm}}^{2}.$$

Given typical values for biological specimens of *ρ* = 1.35 × 10^3^ kg m^−3^ and *E*_m_ = 35 eV = 5.6 × 10^−18^ J [73], and using carbon, which has an atomic number *Z* = 6 and atomic mass of *A* = 12.01, the mean free path at an electron energy of 300 keV is approximately *λ_i_* ≈ 237 nm.

The isotropic *B* factor can then be written in terms of the electron dose as *B* = 8*π*^2^*D*_E_*S*_E_, where *S*_E_ = 1.75 *S*_AD_ = 0.021 A^2^/e^−^. This value is broadly consistent with experimental observations [39] determined the beam damage using purple membrane crystals and reported that the *B* factor increased by 7 A^2^ for every 1 e^−^/A^2^ of exposure at 300 keV. This would give a sensitivity coefficient of *S*_E_ = 0.089 A^2^/e^−^. The model is also broadly consistent with the *B* factors observed and estimated in the Bayesian polishing algorithm implemented in RELION [40] where, for a Ribosome dataset, the total dose of 16 e^−^/A^2^ resulted in a final *B* factor of approximately 90, a β-Galactosidase dataset with a total dose of 45 e^−^/A^2^ resulted in a final *B* factor of approximately 160, and a γ-Secretase dataset with a total dose of 40 e^−^/A^2^ resulted in a final *B* factor of approximately 130. This would correspond to sensitivity coefficients of *S*_E_ = 0.071 A^2^/e^−^, *S*_E_ = 0.048 A^2^/e^−^ and *S*_E_ = 0.041 A^2^/e^−^ respectively.
